# Effect of cold environments on technical performance and perceived workload and stress during advanced medical procedures: a randomized controlled simulation study

**DOI:** 10.1186/s13049-025-01373-8

**Published:** 2025-07-01

**Authors:** Giulia Roveri, Lorenzo Gamberini, Erika Borotto, Frederik Eisendle, Luigi Festi, Hermann Brugger, Giacomo Strapazzon, Simon Rauch, EMEX investigators

**Affiliations:** 1 Department of Medicine, University of Padova, Via Giustiniani 2, Padova, 35128 Italy; 2 Eurac Research, Institute of Mountain Emergency Medicine, Via Ipazia 2, 39100 Bolzano, Italy; 3 Department of Anesthesia and Intensive Care Medicine, “F. Tappeiner” Hospital Merano, Merano, Italy; 4 Department of Intensive Care and Prehospital Emergency, Emergency Department, Maggiore Hospital, Bologna, Italy; 5 Anesthesia and General and Neurosurgical Intensive Care, Di Circolo University Hospital, Varese, Italy; 6 Department of Anaesthesiology and Intensive Care Medicine, Medical University of Innsbruck, Innsbruck, Austria; 7 Department of General, Emergency and Transplant Surgery, Di Circolo University Hospital, Varese, Italy; 8 Corpo Nazionale Soccorso Alpino E Speleologico, National Medical School, (CNSAS SNaMed), Milano, Italy

## Abstract

**Background:**

Advanced medical procedures in prehospital settings are often performed in hostile environments, where cold temperatures may impair manual and cognitive performance. Although such procedures are essential in mountain rescue missions, the effects of cold conditions on their execution and associated workload and stress are unknown.

**Objective:**

This randomized controlled simulation study evaluated differences in performance, perceived workload, and stress during the execution of three advanced emergency medical procedures under cold (− 20 °C) versus control (+ 20 °C) ambient temperatures. Additionally, the study examined the influence of operator experience on these outcomes.

**Methods:**

Thirty-six members of the International Medical Commission for Alpine Rescue participated in a crossover study conducted at the terraXcube environmental simulator in Bolzano, Italy. Participants performed orotracheal intubation via videolaryngoscopy (OTI-VLS), mini-thoracostomy, and front-of-neck airway (FONA) procedures under both temperature conditions. Time to procedure completion, number of attempts, and perceived workload and stress (using the NASA Task Load Index and Visual Analogue Scale) were measured. Operators were categorized into high or low experience groups based on self-reported prior procedure frequency.

**Results:**

Time to complete the procedures tended to be longer in cold conditions for all procedures, with the largest difference observed for OTI-VLS (14 s, *p* = 0.076). Success rates exceeded 90% on the first attempt under both conditions. Perceived workload and stress increased significantly in cold environments across all procedures, especially for less experienced participants. Experienced operators completed OTI-VLS and mini-thoracostomy significantly faster and reported lower stress and workload levels compared to their less experienced counterparts.

**Conclusions:**

While cold environments had low impact on procedural time, they significantly increased perceived workload and stress among rescue personnel. Experience mitigated these effects, emphasizing the importance of tailored training programs to enhance both technical and non-technical skills in challenging conditions. While this study has explored the impact of temperature, it would be valuable to investigate how other environmental factors, such as wind and rain, might affect clinical actions.

**Supplementary Information:**

The online version contains supplementary material available at 10.1186/s13049-025-01373-8.

## Background

Advanced medical procedures in hostile environments pose significant challenges due to harsh conditions and limited resources. [1–7] Despite these difficulties, performing such procedures on site is often essential. Advanced airway management is performed on-site in over one-third of severely injured trauma patients during mountain rescues [8], highlighting the frequent need for advanced interventions in these settings. However, performing advanced medical procedures on site can extend prehospital time, which correlates with higher mortality rates among trauma patients [9]. On-scene orotracheal intubation adds approximately 10 min to a HEMS (Helicopter Emergency Medical Service) mission [10], and each additional 10 min of prehospital time increases the odds of death by 9% [9].

Harsh environmental conditions could compromise the performance of advanced medical procedures and further extend prehospital time. During HEMS mountain rescue missions, rescue personnel often face cold temperatures, [11] which can impair both manual [12, 13] and cognitive performance [14], potentially affecting the execution of medical procedures. Cold environments increase the physical and mental demands on rescuers, [15] raising the risk of performance errors [16–20].

Despite this knowledge, there is a lack of literature investigating whether the time required to perform advanced medical procedures significantly differs under harsh cold conditions compared to comfortable ambient temperatures. This includes both the technical performance of the procedures as well as the workload and stress perceived by the operators. [21–23] Moreover, the impact of rescue personnel’s experience on the performance of advanced medical procedures, as well as on workload and stress levels, remains unclear.

The primary aim of this randomized controlled simulation study was to evaluate differences in performance when executing three advanced emergency medical procedures at cold ambient temperature (− 20 °C) in a controlled environment versus control ambient temperature (+ 20 °C).

Secondary aims included assessing the perceived workload and stress of the operators during the procedures, as well as investigating how the rescue personnel’s prior experience with these advanced medical procedures influences both performance (primary outcome) and perceived workload and stress (secondary outcomes).

## Methods

This randomized controlled simulation study was conducted at the terraXcube, an environmental simulator of Eurac Research (Bolzano, Italy), in October 2023. The study was approved by the Ethics Institutional Review Board for Clinical Studies of Bolzano (protocol number 80–2023) and registered at clinicaltrials.gov (NCT06640595). The study was conducted in accordance with Good Clinical Practice guidelines and followed the Declaration of Helsinki guidelines. All volunteers gave informed consent to participate in the study. The study used a randomized controlled crossover design and included three advanced medical procedures: orotracheal intubation using videolaryngoscopy (OTI-VLS), mini-thoracostomy with chest drain insertion (mini-thoracostomy), and front-of-neck airway (FONA) with the Scalpel-Bougie technique [24].

### Study participants

Thirty-six members of the International Medical Commission for Alpine Rescue (ICAR MedCom) participated in the study. Participant characteristics were collected via an online survey before the study commenced (Table 1). The following details were recorded: age, sex, occupation (physician, nurse, paramedic), medical specialty (Anesthesiology and Intensive Care Medicine, Emergency Medicine, General Practitioner or Internal Medicine, others), and years of experience in both healthcare (including training/residency) and mountain rescue.

**Table 1:** Characteristics of the 36 study participants

Age, median [IQR]	46 [37, 57]
Female sex, n (%)	15 (41%)
Occupation, n (%)	
* Nurse*	2 (6%)
* Paramedic*	1 (3%)
* Physician*	33 (92%)
Anesthesiology and intensive care	12 (30%)
Emergency medicine	41 (41%)
General practitioner or Interal medicine	4 (12%)
others	3 (9%)
Years of work experience in healthcare, median [IQR]	20 [9, 30]
Years of work experience in mountain rescue, median [IQR]	10 [1, 15]
Experience level with advanced medical procedures, n (%)	
* Orotracheal intubation using videolaryngoscopy*	
< 50 times (low level of experience)	21 (58%)
$\underline { > }$ 50 times (high level of experience)	15 (41%)
*Mini*-*thoracostomy with chest drain in sertion*	
< 5 times (low level of experience)	23 (64%)
$\underline { > }$ 5 times (high level of experience)	13 (36%)
*Front of Nect Access*	
< 5 times (low level of experience)	17 (47%)
$\underline { > }$ 5 times (high level of experience)	19 (53%)

IQR: Interquartile range

Participants were also asked to report their experience level with the three advanced medical procedures in the study. They were categorized as having high or low levels of experience based on the self-reported number of procedures performed in their careers, irrespective of whether they were conducted in austere conditions or in-hospital settings. A high level of experience was defined as ≥ 50 OTI-VLS procedures on patients (as previously reported [25]), ≥ 5 mini-thoracostomies on manikins, and ≥ 5 FONA procedures on manikins. Low experience was categorized as < 50 OTI-VLS, < 5 mini-thoracostomies, and < 5 FONA.

### Study design and measurements

Thirty minutes before the experimental session, participants were familiarized with the study materials (videolaryngoscope, scalpel, bougie, chest tube kit and manikin models) and completed a 20-min training session for each advanced procedure on dedicated manikins. These training sessions were not included in the study data. In the cold environment, participants wore their winter mountain rescue clothing provided by their organization. In the control environment, procedures were performed using nitrile medical gloves, while in the cold environment, participants could choose to wear additional winter gloves. They could interrupt the study at any time and be moved to a warm room.

Following the familiarization session, each participant, according to the randomization list, performed the three advanced medical procedures (OTI-VLS, mini-thoracostomy, FONA) twice: once under control environmental conditions (+ 20 °C; no wind; relative humidity 30 ± 10%) and once under cold environmental conditions (− 20 °C; no wind; relative humidity 70 ± 10%) (Supplement Fig. 1). Before starting the medical procedure, participants were exposed to the respective environmental condition for 5 min. Data were collected via video recordings, which were assessed by independent evaluators after the tests. Evaluators were not blinded to the temperature conditions, as participants wore different clothing based on the environmental condition. The time to complete each procedure (in seconds) and the total number of attempts were recorded.

For OTI-VLS, the C-MAC videolaryngoscope with a Macintosh blade was used (Karl Storz SE & Co. KG Germany). The procedure began when the participant first took in hand the videolaryngoscope and ended when the endotracheal tube (ETT) was placed into the manikin’s trachea. Correct ETT placement was confirmed by inflating the lungs with a ventilation bag and observing visible lung expansion in the mannikin. An “attempt” was defined as a single insertion of the laryngoscope blade, followed by either the insertion of the ETT or the insertion of a bougie and subsequent placement of the ETT [26].

For the mini-thoracostomy procedure, the start was defined as the moment when the participant first touched the mannikin and the procedure ended when the pleural space was opened. Verification of pleural space entry was conducted by the simulation supervisor. An “attempt” was defined as any attempt to open the pleural space using forceps.

For the FONA procedure, the start was marked when the participant first touched the mannikin, and the end was defined when the ETT was placed into the trachea. Correct placement was verified by bag ventilations through the ETT and defined as visible expansion of both lungs. An “attempt” was defined as single insertion attempt of the ETT through the cricothyrotomy.

Immediately after completing the procedures, participant’s perceived stress levels and self-rated performance were measured using a Visual Analogue Scale (VAS), consisting of a 10 cm line with two end points (0 = no stress and 10 = maximal stress; 0 = bad performance and 10 = good performance).

Concurrently, the perceived task workload was assessed through the NASA Task Load Index (NASA-TLX) self-assessment tool. [27–31] The index consists of 6 subscales: mental demand, physical demand, temporal demand, performance, effort, and frustration. Each subscale is rated on a 20-point scale (0 = low, 20 = high). VAS and NASA-TLX subscales data were transcribed from paper by two independent research team members (GR and CD). All data were entered in Microsoft Excel database.

The primary outcome of the study was the time taken to perform the procedures at -20 °C compared to control ambient temperature. Secondary outcomes included the number of attempts for successful completion, as well as the perceived workload and stress of the operators during the procedures. Both primary and secondary outcomes were compared between operators with high and low experience levels.

### Statistical analysis

The sample size calculation for the OTI-VLS was based on the study by Mallick et al. [32], in which the standard deviation for time to intubation with a video laryngoscope was 10 s. Assuming 10 s as the standard deviation of the time difference for intubation in a control and cold environment, a sample size of 13 participants was determined to detect a difference of 10 s, with a Type I error of 0.05 and a statistical power of 95%. Assuming a 20% dropout rate, the total sample size was adjusted to 16 participants.

For mini-thoracostomy and FONA, no prior studies are available on which to base the sample size calculation. The sample size calculation was set to meet pilot study criteria, requiring a minimum of 12 subjects to ensure reliable estimates of mean and variance [33].

A random allocation sequence was generated with R software (R Core Team, 2023).

The general characteristics of the participants were summarized using summary statistics. Continuous variables were reported as means and standard deviations (SD) when normally distributed (assessed by QQ plots) or as medians and interquartile ranges (IQR) for non-normally distributed data. Categorical variables were presented as absolute and relative frequencies.

To compare intraindividual data recorded in cold and normal temperatures, continuous variables were analyzed using the Paired-Samples t-test when the normality assumption was satisfied. For non-normally distributed data, the Wilcoxon Signed-Rank test was used. Categorical variables were analyzed using McNemar’s Test, suitable for binary and nominal data.

To evaluate the effect of the experience level on the time required to complete each procedure, perceived workload, and stress, as well as the interaction effect between the experience level and environmental temperature (difference-in-difference), a bivariate ANOVA for repeated measures was conducted. When the assumption of normality was not met, the underlying distribution was verified together with the mean cell frequencies [34] and, if appropriate, an Aligned Ranks Transformation [35] was applied prior to the ANOVA.

Finally, to account for multiple comparisons, *p*-values were adjusted to control the false discovery rate (FDR) at 5% using the Benjamini–Hochberg procedure, [36] thereby reducing the likelihood of type I errors in hypothesis testing.

All the analyses were conducted using R version 4.4.1.

## Results

### Participants

A total of 36 emergency health care professionals participated in the study. Their characteristics, including experience level with the three advanced medical procedures, are shown in Table 1.

### Time required and number of attempts for successful procedure completion

**Fig. 1:**
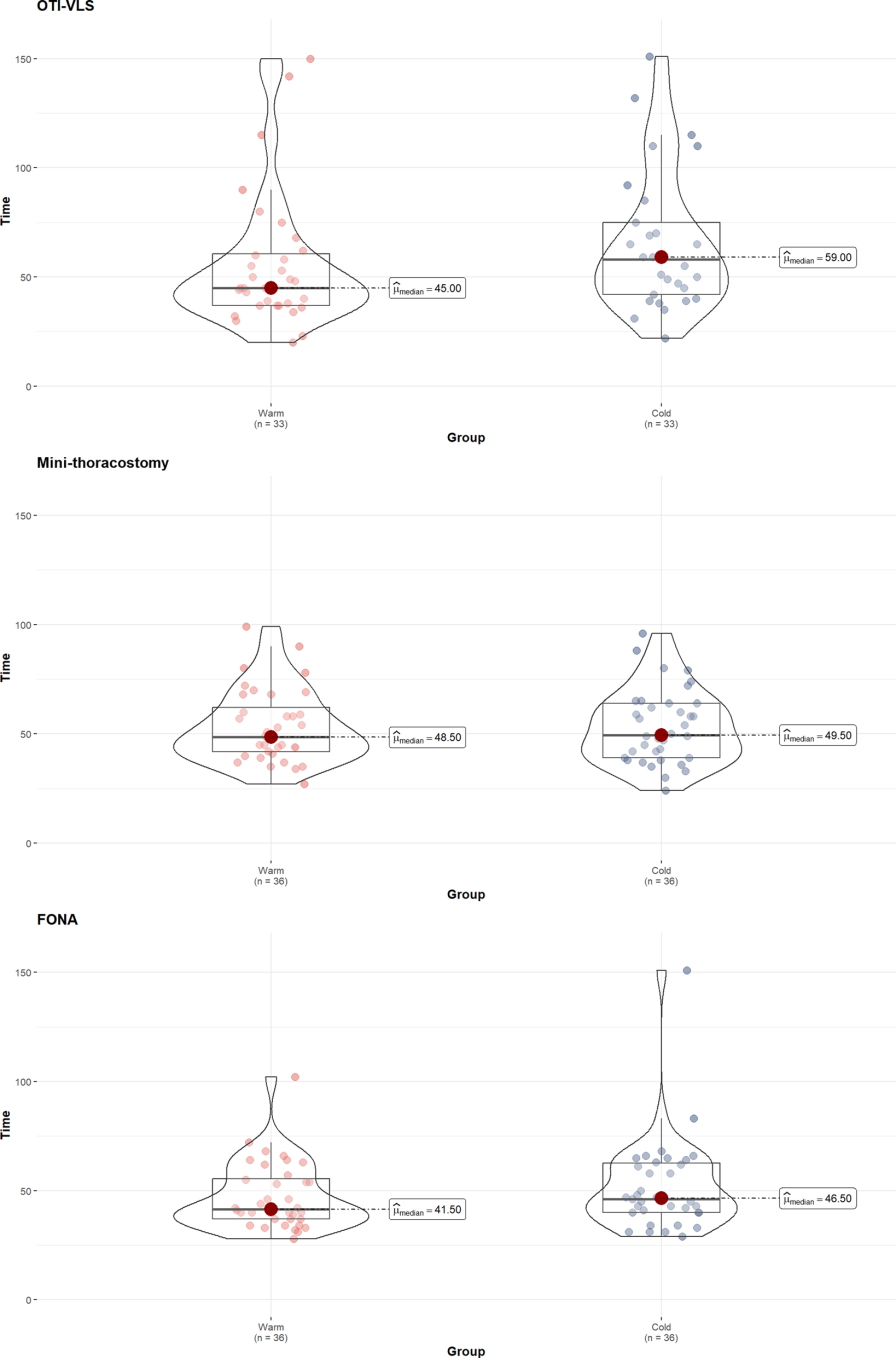
Time to complete each procedure (in seconds) in control and cold environments for the overall study population. Abbreviations: OTI-VLS: orotracheal intubation using videolaryngoscopy. Mini-thoracostomy: mini-thoracostomy with chest drain insertion. FONA: front-of-neck airway with the Scalpel-Bougie technique. Notes: Colored points are single observations. Boxplots show medians, interquartile ranges, and whiskers indicate min and max values. The violin plot details the distribution of observed times, with wider sections indicating higher frequency

The time required to perform the procedures tended to be longer under cold conditions compared to control conditions (Fig. 1): OTI-VLS 59.0 vs 45.0 s (*p* = 0.076), mini-thoracostomy 49.5 vs 48.5 s (*p* = 0.804) and FONA 46.5 vs 41.5 s (*p* = 0.161).

Overall, the success rate exceeded 90% for all maneuvers on the first attempt under both, cold and control conditions. The number of attempts is outlined in Supplement Table 1.

### Workload and Stress

**Fig. 2:**
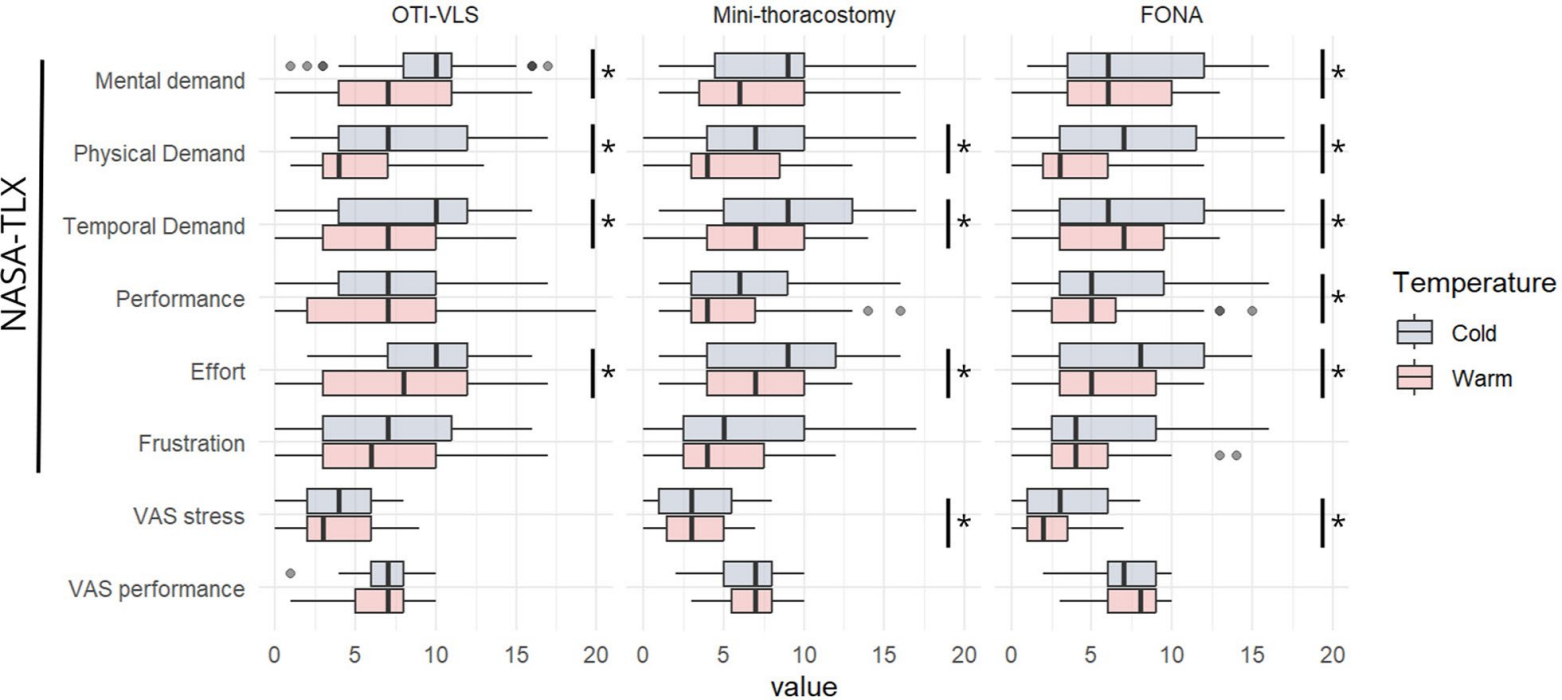
Levels of workload and stress across the procedures in control and cold environments for the overall study population. Abbreviations: OTI-VLS: orotracheal intubation using videolaryngoscopy. Mini-thoraco: mini-thoracsotomy with chest drain insertion. FONA: front-of-neck airway with the Scalpel-Bougie technique. NASA-TLX: Nasa Task Load Index; VAS: Visual Analogue Scale. Notes: Boxplots show medians and interquartile ranges; whiskers indicate min and max values; points are outliers. * *p* < 0.05 (Wilcoxon Signed-Rank test)

Self-reported workload and stress levels in cold and control conditions are depicted in Fig. 2.

For OTI-VLS, NASA-TLX scores for median mental, physical, and temporal demands, as well as effort were significantly higher under cold conditions compared to control conditions: 10 vs 7 (*p* = 0.014), 7 vs 4 (*p* = 0.005), 10 vs 7 (*p* = 0.032), and 10 vs 8 (*p* = 0.024), respectively. However, self-rated VAS stress and performance scores did not differ significantly between control and cold conditions.

For mini-thoracostomy, median physical and temporal demands, as well as effort significantly increased in cold compared to control conditions: 7 vs 4 (*p* = 0.022), 9 vs 7 (*p* = 0.045) and 9 vs 7 (*p* = 0.032), respectively. VAS stress scores were slightly higher in cold conditions (3.0 vs 2.5, *p* = 0.047), while VAS performance scores remained unchanged.

For FONA, all main components of NASA-TLX, except for temporal demand and frustration, were significantly higher in cold temperature. Additionally, VAS stress scores were higher under cold conditions (3.0 vs 2.0, *p* = 0.006).

All pairwise comparisons between cold and control environments are presented in the Supplement Table 1. Participants consistently reported higher physical and temporal demands, effort, and stress (except for OTI-VLS) under cold conditions across all procedures, indicating an increased perception of workload and stress.

### Effect of experience level on procedure time and perceptions of workload and stress

**Fig. 3:**
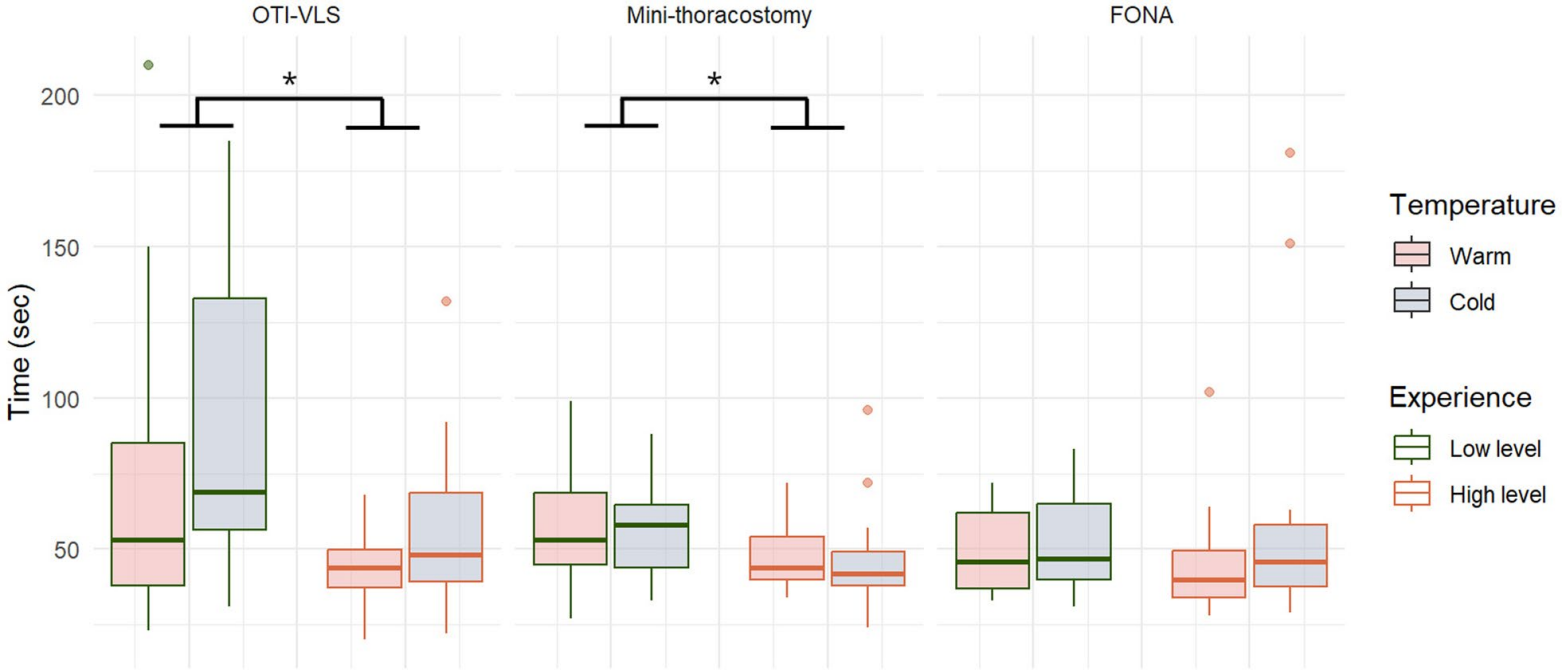
Time to complete each procedure (in seconds) in control and cold environments. Comparison between high- and low-level experience clinicians. Abbreviations: OTI-VLS: orotracheal intubation using videolaryngoscopy. Mini-thoraco: mini-thoracostomy with chest drain insertion. FONA: front-of-neck airway with the Scalpel-Bougie technique. Notes: Boxplots show medians and interquartile ranges; whiskers indicate min and max values; points are outliers. * p < *0.05 for Experience effect in bivariate ANOVA. The interaction between Experience and Temperature showed no significant effect on any variables*

For both OTI-VLS and mini-thoracostomy, participants with low experience level took significantly longer than those with high experience level (*p* = 0.018 and *p* = 0.035, respectively) (Fig. 3). In contrast, there was no significant difference in the time required for FONA between low-level and high-level experience participants.

Participants’ perceptions of workload and stress based on their level of experience are shown in Supplement Fig. 4. For OTI-VLS, participants with low experience reported a significantly higher VAS stress compared to those with high experience (*p* = 0.048). For mini-thoracostomy, less experienced participants reported higher median mental (*p* < 0.001), physical (*p* = 0.018), and temporal (*p* = 0.018) demands compared to their more experienced counterparts, along with increased NASA-TLX subscale scores for performance (*p* = 0.003), as well as elevated VAS stress scores (*p* = 0.018). No experience-related differences were found for FONA.

The interaction [difference-in-difference] between temperature (cold vs control environment) and experience (low vs high-level) did not significantly impact the time needed to complete the procedures, the components of NASA-TLX, or the perceived stress and performance (Supplement Table 2).

## Discussion

This randomized controlled simulation study aimed to investigate the impact of cold environmental conditions on the execution of advanced medical procedures, along with the associated workload and stress perceived by medical personnel. Our findings show that, while the time required to perform the procedures and the number of attempts for successful completion were similar in both cold and control environments, the workload and stress levels were perceived to be generally higher in cold conditions. Additionally, operator experience was found to be a significant factor in procedural time and perceived stress during OTI-VLS and minithoracostomy, under both cold and control temperature conditions.

### Impact of cold environment on procedure time and attempts

The primary outcome of the study was the time required to complete each procedure under cold versus control ambient temperatures. Although the number of attempts did not differ between the control and cold environments, the data suggest a trend toward increased time requirements under cold conditions for all three procedures. This was most pronounced for OTI-VLS, with a 14-s delay. While this might seem clinically insignificant, in a real-world prehospital setting—where other environmental factors such as wind or poor visibility may be more extreme—this time difference could have clinically relevant implications. Critically ill patients can desaturate rapidly following rapid sequence induction, [37] making even small-time delays clinically meaningful in terms of oxygenation and patient outcomes. Furthermore, while not measured in this study, it is likely that the preparatory phase—such as gathering and preparing the needed materials—would also take longer in cold conditions, further extending the total procedure time. From a practical standpoint, the use of chemical hand warmers for rescuers, as well as the use of shelters during rescues in austere conditions, can improve physical comfort during medical procedures, thereby enhancing patient care. [38]

### Perceived workload and stress

The workload and stress experienced by medical personnel operating in complex environments are often underestimated. [39] In our cohort, subjective ratings across all six NASA-TLX measures, along with stress parameters measured via VAS, revealed significant increases during procedures in cold conditions. For procedures like OTI-VLS, participants reported notably higher mental, physical, and temporal demands, along with increased effort in cold environments. Similarly, mini-thoracotomy and FONA procedures showed heightened physical and temporal demands, emphasizing the substantial effort required in adverse conditions.

These findings support previous studies [12–14] indicating that cold environments increase stress and workload, even when technical performance is not severely compromised. Elevated stress in unfamiliar or unfavorable working conditions is well-documented. [40, 41] Prehospital care often occurs in uncontrolled, high-stress settings, [42] making it essential for healthcare professionals to be adequately prepared for the additional cognitive and physical burdens posed by cold conditions. Incorporating simulation-based training under harsh environmental conditions can play a critical role in equipping professionals to effectively manage these challenges, enhancing both technical and non-technical skill proficiency and resilience in real-world scenarios [43].

### Effect of experience on performance and perceptions

Operator experience, assessed by the self-reported number of procedures performed during their careers, significantly impacted both procedure times and perceived workload. Participants with fewer prior procedures took significantly longer to complete OTI-VLS and mini-thoracostomy, especially in cold environments. These findings suggest that experience can mitigate some of the negative effects of cold on procedural performance, potentially due to greater familiarity with the procedures and better ability to regulate stress under pressure.

Additionally, less experienced participants consistently reported higher mental and physical demands, as well as higher stress levels, compared to their more experienced counterparts. This finding emphasizes the importance of training and experience in prehospital settings, especially in challenging environments like cold climates [43]. Structured training programs that focus on enhancing both technical and non-technical skills [44] as well as stress management [45] in simulated scenarios in realistic environments may help less experienced practitioners perform better under harsh conditions.

### Strengths and limitations

A key strength of the study was the use of a climate-controlled chamber, which standardized environmental conditions, minimizing bias compared to in-field studies. The crossover design, with participants serving as their own controls, and the randomized order further enhanced reliability. The cohort was highly representative, consisting of professionals in mountain rescue with expertise in advanced medical procedures.

On the other side, the chamber study could not consider adverse conditions in real scenarios, where additional technical, environmental, and human factors could negatively impact emergency treatment, as well as the complex interplay of actions and outcomes. Furthermore, the use of manikin models, rather than real patients, limits the assessment of issues like equipment fogging, and the focus on only three procedures may reduce generalizability. Additionally, we did not analyze the potential differential effects of cold exposure by sex, as our study was not sufficiently powered to detect sex-based differences. The study focuses on advanced medical procedures, which are intended to be usually performed by highly trained professionals. We also did not perform a subgroup analysis due to the small sample sizes among paramedics (n = 1) and nurses (n = 2), compared to physicians (n = 33). Therefore, the findings cannot be directly translated to emergency personnel with specific training or experience levels. Finally, the small sample size may have limited the detection of more nuanced differences in some outcomes, especially in the differences in time required to complete the procedures under cold versus control ambient temperatures. However, the study’s findings remained robust even after adjusting for multiple comparisons to control the false discovery rate.

## Conclusions

This study highlights the significant increase in perceived workload and stress when performing advanced medical procedures in cold environments, despite a non-significant trend in procedural time. Operator experience plays a crucial role in mitigating the negative effects of cold conditions. These findings underscore the importance of tailored training to prepare emergency medical personnel for the unique challenges of providing care in hostile environments, where cold temperatures can amplify both cognitive and physical demands. While this study has explored the impact of temperature, it would also be valuable to investigate how other environmental factors, such as wind and rain, might affect clinical actions. Moreover, future research could explore adaptive strategies to address the challenges posed by cold environments during advanced medical procedures.

## Supplementary Information

Supplementary Material 1.

## Data Availability

The datasets used and/or analysed during the current study are available from the corresponding author on reasonable request.
