# Supplementary material for: Effect of cold environments on technical performance and perceived workload and stress during advanced medical procedures: a randomized controlled simulation study
Source: Scand J Trauma Resusc Emerg Med. 2025 Jul 1;33:113. doi: 10.1186/s13049-025-01373-8 (PMC12211314; doi:10.1186/s13049-025-01373-8)
Supplement: Supplementary file 1 — Supplementary Material 1. [file 13049_2025_1373_MOESM1_ESM.docx]

**Electronic Supplementary Material**

| Difficult Orotracheal Intubation | Warm (+20 °C) | Cold (-20°C) | p | p FDR |
| --- | --- | --- | --- | --- |
| Total number of attempts to success (median [IQR]) | 1.00 [1.00, 1.00] | 1.00 [1.00, 1.00] | 0.660 | 0.741 |
| Time required - sec - (median [IQR]) | 45.00 [37.00, 62.00] | 59.00 [45.00, 110.00] | 0.038 | 0.079 |
| Mental demand (median [IQR]) | 7.00 [4.00, 11.00] | 10.00 [8.00, 11.00] | 0.002 | 0.014 |
| Physical Demand (median [IQR]) | 4.00 [3.00, 7.00] | 7.00 [4.00, 12.00] | 0.000 | 0.005 |
| Temporal Demand (median [IQR]) | 7.00 [3.00, 10.00] | 10.00 [4.00, 12.00] | 0.011 | 0.032 |
| Performance (median [IQR]) | 7.00 [2.00, 10.00] | 7.00 [4.00, 10.00] | 0.652 | 0.741 |
| Effort (median [IQR]) | 8.00 [3.00, 12.00] | 10.00 [7.00, 12.00] | 0.007 | 0.024 |
| Frustration (median [IQR]) | 6.00 [3.00, 10.00] | 7.00 [3.00, 11.00] | 0.258 | 0.333 |
| VAS stress (median [IQR]) | 3.00 [2.00, 6.00] | 4.00 [2.00, 6.00] | 0.249 | 0.333 |
| VAS performance (median [IQR]) | 7.00 [5.00, 8.00] | 7.00 [6.00, 8.00] | 0.687 | 0.741 |
| Minithoracotomy and Chest Tube Placement | **Warm (+20 °C)** | **Cold (-20°C)** | **p** | **p FDR** |
| Total number of attempts to success (median [IQR]) | 1.00 [1.00, 1.00] | 1.00 [1.00, 1.00] | 0.346 | 0.429 |
| Time required - sec - (median [IQR]) | 48.50 [41.75, 62.00] | 49.50 [39.00, 64.00] | 0.778 | 0.804 |
| Procedure correct (0=no; 1= si) = 1 (%) | 33 (91.7) | 34 (94.4) | 1.000 | 1.000 |
| Mental demand (median [IQR]) | 6.00 [3.50, 10.00] | 9.00 [4.50, 10.00] | 0.083 | 0.152 |
| Physical Demand (median [IQR]) | 4.00 [3.00, 8.50] | 7.00 [4.00, 10.00] | 0.004 | 0.022 |
| Temporal Demand (median [IQR]) | 7.00 [4.00, 10.00] | 9.00 [5.00, 13.00] | 0.020 | 0.047 |
| Performance (median [IQR]) | 4.00 [3.00, 7.00] | 6.00 [3.00, 9.00] | 0.099 | 0.161 |
| Effort (median [IQR]) | 7.00 [4.00, 10.00] | 9.00 [4.00, 12.00] | 0.010 | 0.032 |
| Frustration (median [IQR]) | 4.00 [2.50, 7.50] | 5.00 [2.50, 10.00] | 0.238 | 0.333 |
| VAS stress (median [IQR]) | 2.50 [1.75, 5.00] | 3.00 [1.00, 5.25] | 0.018 | 0.047 |
| VAS performance (median [IQR]) | 7.00 [5.75, 8.00] | 7.00 [5.00, 8.00] | 0.178 | 0.276 |
| FONA | **Warm (+20 °C)** | **Cold (-20°C)** | **p** | **p FDR** |
| Total number of attempts to success (median [IQR]) | 1.00 [1.00, 1.00] | 1.00 [1.00, 1.00] | 0.693 | 0.741 |
| Time required - sec - (median [IQR]) | 41.50 [37.00, 55.50] | 46.50 [40.00, 63.25] | 0.098 | 0.161 |
| Mental demand (median [IQR]) | 6.00 [3.50, 10.00] | 6.00 [3.50, 12.00] | 0.006 | 0.024 |
| Physical Demand (median [IQR]) | 3.00 [2.00, 6.00] | 7.00 [3.00, 11.50] | 0.000 | 0.001 |
| Temporal Demand (median [IQR]) | 7.00 [3.00, 9.50] | 6.00 [3.00, 12.00] | 0.007 | 0.024 |
| Performance (median [IQR]) | 5.00 [2.50, 6.50] | 5.00 [3.00, 9.50] | 0.023 | 0.050 |
| Effort (median [IQR]) | 5.00 [3.00, 9.00] | 8.00 [3.00, 12.00] | 0.001 | 0.006 |
| Frustration (median [IQR]) | 4.00 [2.50, 6.00] | 4.00 [2.50, 9.00] | 0.044 | 0.086 |
| VAS stress (median [IQR]) | 2.00 [1.00, 3.50] | 3.00 [1.00, 6.00] | 0.001 | 0.006 |
| VAS performance (median [IQR]) | 8.00 [6.00, 9.00] | 7.00 [6.00, 9.00] | 0.212 | 0.313 |

Supplement Table 1 – Differences in number of attempts, time and perception in warm and cold environments

Notes: False Discovery Rate (FDR) procedure-adjusted p values are shown.

Abbreviations: IQR – interquartile range; VAS – Visual analog scale

Supplement Table 2 – Experience level related differences in high and cold environments

| Procedure | Variable | Warm – Low-level experience | Cold - Low-level experience | Warm - High-level experience | Cold - High-level experience | p Exp | p Exp FDR | p Exp*Group | p Exp*Group FDR |
| --- | --- | --- | --- | --- | --- | --- | --- | --- | --- |
| Difficult Orotracheal Intubation | Time - sec - IQR | 53.00 [38.00, 85.00] | 69.00 [56.50, 133.00] | 44.00 [37.25, 49.75] | 48.00 [39.25, 68.75] | 0.004 | 0.018 | 0.406 | 0.992 |
| Difficult Orotracheal Intubation | NASA - Mental – IQR | 8.50 [4.25, 11.75] | 10.00 [8.25, 12.75] | 5.00 [3.00, 10.00] | 9.00 [5.00, 10.00] | 0.126 | 0.200 | 0.815 | 0.992 |
| Difficult Orotracheal Intubation | NASA – Physical – IQR | 4.50 [3.00, 9.25] | 9.00 [6.25, 13.00] | 4.00 [3.00, 5.00] | 5.00 [4.00, 8.50] | 0.026 | 0.063 | 0.329 | 0.992 |
| Difficult Orotracheal Intubation | NASA – Temporal – IQR | 7.00 [4.00, 10.00] | 10.00 [6.00, 12.75] | 6.00 [2.25, 8.00] | 5.50 [4.00, 10.75] | 0.071 | 0.137 | 0.950 | 0.992 |
| Difficult Orotracheal Intubation | NASA – Performance – IQR | 8.00 [3.00, 13.75] | 8.50 [4.25, 10.75] | 5.00 [1.25, 7.00] | 4.00 [4.00, 9.00] | 0.067 | 0.137 | 0.774 | 0.992 |
| Difficult Orotracheal Intubation | NASA – Effort – IQR | 6.50 [3.25, 12.75] | 12.00 [10.00, 14.00] | 8.00 [2.25, 10.00] | 9.00 [4.25, 10.00] | 0.097 | 0.163 | 0.633 | 0.992 |
| Difficult Orotracheal Intubation | NASA – Frustration – IQR | 5.50 [3.00, 8.00] | 8.50 [6.00, 12.75] | 5.00 [1.25, 9.25] | 4.00 [2.00, 8.75] | 0.067 | 0.137 | 0.310 | 0.992 |
| Difficult Orotracheal Intubation | VAS – stress – IQR | 3.00 [2.00, 6.00] | 5.00 [3.00, 6.75] | 2.00 [0.25, 4.50] | 2.50 [1.25, 4.75] | 0.016 | 0.048 | 0.965 | 0.992 |
| Difficult Orotracheal Intubation | VAS – performance – IQR | 7.00 [5.00, 8.00] | 6.00 [5.25, 7.00] | 7.00 [5.00, 8.75] | 7.50 [6.00, 8.00] | 0.230 | 0.311 | 0.829 | 0.992 |
| Minithoracotomy and Chest Tube Placement | Time - sec - IQR | 53.00 [45.00, 68.50] | 58.00 [44.00, 64.50] | 44.00 [40.00, 54.00] | 42.00 [38.00, 49.00] | 0.011 | 0.036 | 0.722 | 0.992 |
| Minithoracotomy and Chest Tube Placement | NASA – Mental – IQR | 6.00 [4.25, 10.00] | 9.00 [5.00, 10.00] | 7.00 [3.00, 10.00] | 7.00 [4.00, 10.00] | 0.000 | 0.000 | 0.707 | 0.992 |
| Minithoracotomy and Chest Tube Placement | NASA – Physical – IQR | 5.00 [3.00, 8.75] | 7.50 [4.25, 10.00] | 4.00 [3.00, 6.00] | 6.00 [4.00, 7.00] | 0.004 | 0.018 | 0.167 | 0.992 |
| Minithoracotomy and Chest Tube Placement | NASA – Temporal – IQR | 7.00 [4.00, 10.00] | 9.50 [4.25, 13.00] | 7.00 [4.00, 12.00] | 9.00 [7.00, 13.00] | 0.005 | 0.018 | 0.655 | 0.992 |
| Minithoracotomy and Chest Tube Placement | NASA – Performance – IQR | 5.00 [3.25, 7.75] | 6.00 [3.00, 9.00] | 4.00 [2.00, 7.00] | 4.00 [3.00, 7.00] | 0.000 | 0.003 | 0.793 | 0.992 |
| Minithoracotomy and Chest Tube Placement | NASA – Effort – IQR | 6.50 [4.25, 9.75] | 8.50 [4.00, 12.00] | 8.00 [4.00, 10.00] | 9.00 [4.00, 11.00] | 0.003 | 0.018 | 0.244 | 0.992 |
| Minithoracotomy and Chest Tube Placement | NASA – Frustration – IQR | 4.00 [3.00, 7.00] | 5.50 [2.25, 9.50] | 4.00 [1.00, 10.00] | 4.00 [3.00, 10.00] | 0.023 | 0.062 | 0.612 | 0.992 |
| Minithoracotomy and Chest Tube Placement | VAS – stress – IQR | 2.00 [1.50, 5.00] | 2.00 [1.00, 5.00] | 3.00 [2.00, 4.00] | 4.00 [3.00, 6.00] | 0.005 | 0.018 | 0.142 | 0.992 |
| Minithoracotomy and Chest Tube Placement | VAS – performance – IQR | 7.00 [5.00, 8.00] | 7.00 [5.00, 8.00] | 8.00 [7.00, 8.00] | 8.00 [7.00, 8.00] | 0.084 | 0.152 | 0.741 | 0.992 |
| Front Of Neck Access | Time - sec - IQR | 46.00 [37.00, 62.00] | 47.00 [40.00, 65.00] | 40.00 [34.00, 49.50] | 46.00 [37.50, 58.00] | 0.166 | 0.249 | 0.796 | 0.992 |
| Front Of Neck Access | NASA – Mental – IQR | 7.00 [4.25, 10.00] | 8.50 [4.00, 10.50] | 5.00 [3.50, 10.00] | 6.00 [3.00, 12.00] | 0.504 | 0.648 | 0.922 | 0.992 |
| Front Of Neck Access | NASA – Physical – IQR | 3.00 [2.00, 6.75] | 8.50 [3.75, 11.25] | 3.00 [2.00, 5.00] | 6.00 [3.00, 10.50] | 0.637 | 0.782 | 0.712 | 0.992 |
| Front Of Neck Access | NASA – Temporal – IQR | 7.00 [2.00, 10.00] | 7.50 [3.75, 11.25] | 4.00 [3.00, 9.00] | 6.00 [3.00, 12.00] | 0.899 | 0.899 | 0.970 | 0.992 |
| Front Of Neck Access | NASA – Performance – IQR | 5.50 [2.75, 6.25] | 5.00 [3.00, 9.25] | 4.00 [2.50, 8.00] | 5.00 [3.50, 10.00] | 0.791 | 0.890 | 0.819 | 0.992 |
| Front Of Neck Access | NASA – Effort – IQR | 7.00 [3.00, 10.00] | 7.00 [3.00, 10.00] | 4.00 [3.00, 7.00] | 10.00 [3.00, 13.00] | 0.774 | 0.890 | 0.199 | 0.992 |
| Front Of Neck Access | NASA – Frustration – IQR | 3.00 [3.00, 6.00] | 4.00 [3.75, 6.75] | 4.00 [2.00, 6.00] | 4.00 [2.00, 10.50] | 0.880 | 0.899 | 0.992 | 0.992 |
| Front Of Neck Access | VAS – stress – IQR | 2.00 [1.00, 3.00] | 3.50 [1.75, 6.00] | 3.00 [1.50, 4.00] | 2.00 [1.00, 6.50] | 0.862 | 0.899 | 0.545 | 0.992 |
| Front Of Neck Access | VAS – performance - IQR | 7.50 [6.00, 8.00] | 7.00 [6.00, 8.25] | 8.00 [6.50, 9.00] | 7.00 [6.00, 9.00] | 0.205 | 0.291 | 0.965 | 0.992 |

Note: p values are related to the two-way ANOVA for repeated measures. p Exp refers to p value of the effect related to the experience level, while p Exp*Group is the p value for the difference in difference related to experience level between cold and warm temperature.

False Discovery Rate (FDR) procedure-adjusted p values are also shown.

Abbreviations: IQR – interquartile range; VAS – Visual analog scale

Supplement Figure 1 – Advanced medical procedures and study setting


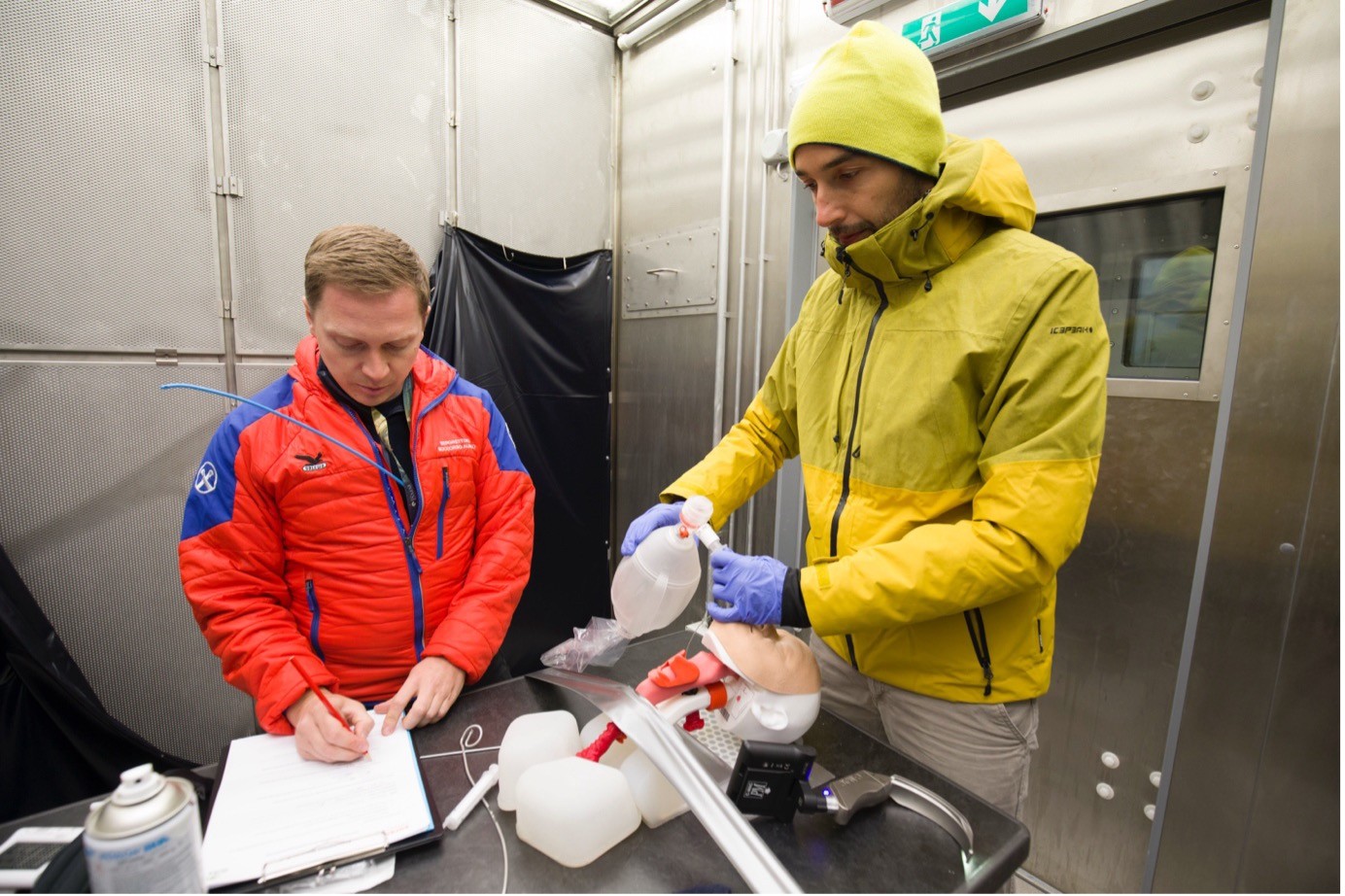


Supplement Figure 2 – Levels of workload and stress across the procedures in warm and cold environments. Comparison between high- and low-level experience clinicians.


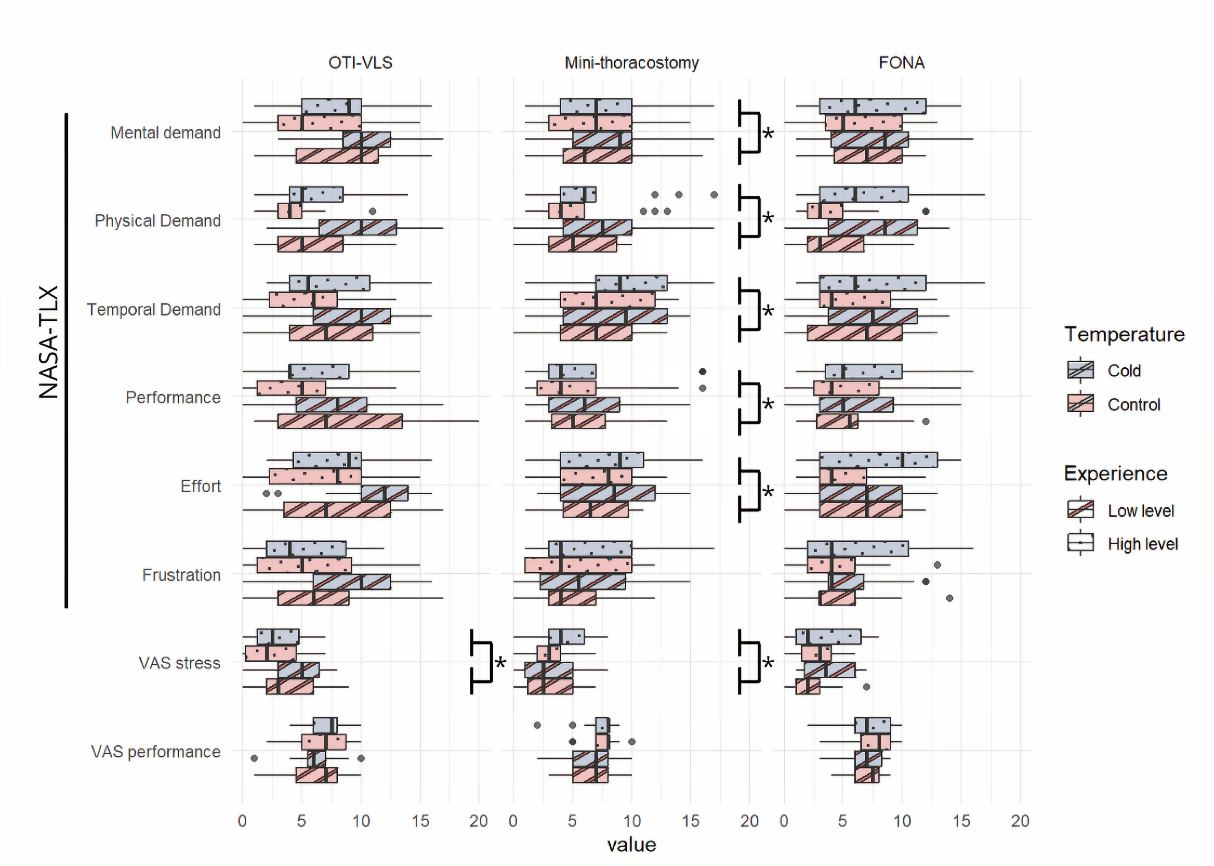


Abbreviations: OTI-VLS: orotracheal intubation using videolaryngoscopy Mini-thoraco: mini-thoracostomy with chest drain insertion. FONA: front-of-neck airway with the Scalpel-Bougie technique. NASA-TLX: Nasa Task Load Index; VAS: Visual Analogue Scale.
Notes: Boxplots show medians and interquartile ranges; whiskers indicate min and max values; points are outliers. * p<0.05 for Experience effect in bivariate ANOVA. The interaction between Experience and Temperature showed no significant effect on any variables.
